# Conclusions and Recommendations

**DOI:** 10.1007/978-3-030-45979-6_7

**Published:** 2020-06-05

**Authors:** Marina Foltea

**Affiliations:** Institute of Law Studies, Polish Academy of Sciences, Warsaw, Poland

## Abstract

Brexit represents an unprecedented moment for the European integration  project. Despite the evident difficulties in assessing the impact of the still ongoing process on the cladenstine activity of illicit tradde, all in all, the potential effects of Brexit on illicit tobacco trade in the UK could be rather modest. One thing which emerges with certain clarity is that just as in many other policy areas, the control of the illicit trade of tobacco by the UK will depend ultemately on the Brexit scenario negotiated with EU. In the event of a leaving the single market, while losing economically on many ends, the UK would retain higher policy flexibilites. While still bound by the ITP, this may mean greater speed and flexibility in learning and improving upon existing global or regional norms in this field to its own benefit.

Brexit represents an unprecedented moment for the European integration  project. Despite the evident difficulties in assessing the impact of the still ongoing process on the cladenstine activity of illicit tradde, all in all, the potential effects of Brexit on illicit tobacco trade in the UK could be rather modest. One thing which emerges with certain clarity is that just as in many other policy areas, the control of the illicit trade of tobacco by the UK will depend ultemately on the Brexit scenario negotiated with EU. In the event of a leaving the single market, while losing economically on many ends, the UK would retain higher policy flexibilites. While still bound by the ITP, this may mean greater speed and flexibility in learning and improving upon existing global or regional norms in this field to its own benefit.

The data presented in this study shows that the UK is a target country for illicit tobacco trade. The country has no significant domestic production (licit or illicit), and is not a transit country for illicit products *en route* to other EU Member States. The focus is therefore mainly on how Brexit may impact illicit trade into the UK. The case of Gibraltar, where illicit products tend to move from the UK territory into (the EU) Spain, represents an exception. Gibraltar already currently enjoys a special status and maintains a rather ‘hard’ border with neighbouring Spain. The *2019 Withdrawal Agreement* foresees a Protocol accompanied by a bilateral *‘Memorandum of Understanding on Tobacco and other Products*’ with Spain, capping the price difference between adjacent territories and fostering cooperation and enforcement. This should limit the risks of an increase of illicit activities related to tobacco targeting the EU market from Gibraltar as a result of Brexit.

More generally, opportunities for smuggling into the UK may arise from an unorderly implementation of its withdrawal from the EU, a scenario that could materialize in case of ‘*Hard Brexit*’. The acceptance of the *2019 Withdrawal Agreement* by the UK Parliament after the December 2019 elections and the start of the transition period (now set to last until the end of 2020) should prevent this. Much will of course depend on the UK’s ability to implement the changes necessary for (licit) trade to be carried out in an orderly manner.

Brexit is not expected to have dramatic effects on the relevant legislation analysed. Most of it is already domestic (UK) legislation and directly applicable EU law will be retained in UK law after exit day. Whether and how in the future the UK will remain aligned to relevant EU legislation depends on several factors. The December elections brought clarity as to the most likely approach to future UK–EU relations. Now that the UK has officially withdrawn on January 31st 2020, the two parties are expected to negotiate an ambitious trade agreement.

Little, however, has been said regarding the unavoidable trade-off between ease of access to the EU markets and autonomy to diverge from EU regulation. This is particularly important for the future application of select aspects of the EU 2014 Tobacco Products Directive (TPD-2), of relevance to the control of tobacco trade. In general however, the past record and the current HMRC and UK Border Force joint national strategy in place (‘*Tackling illicit tobacco: From leaf to light*’) suggests that the country will maintain its strict tobacco regulations.

Two additional aspects that may have an indirect impact on illicit trade also depend on the final form of Brexit. These are import tariffs and thresholds for personal allowances for tobacco products. In an environment of elevated prices (currently among the highest in the world), the adoption of excessively (trade or fiscal) restrictive measures on tobacco may indirectly strengthen the demand for illicit products. The option of levying import tariffs on tobacco products originating from the EU would only be available in case of ‘*Hard Brexit*’. Thus, the materialisation of this scenario would grant the UK independent decision-making powers to establish its desired level of import duties. The complex economic constelation created by Brexit (and pegorated by the Covid-19 crisis) may push the UK into raising its import duties or internal taxes on tobacco. Under the three other Brexit scenarios, including the case of conclusion of a trade agreement, the parties would exchange tobacco products without applying import duties. If the UK remains part of the single market, it would have to abide by the EU minimum excise requirements (in this scenario the UK is till free to raise its excise given the concern of the EU legislation with minimum levels). Under all scenarios analysed, the UK would have the latitude to adopt the tobacco personal allowances threshold it wishes, without consideration for EU imposed minimum levels.

The analysis above also suggests that cooperation with EU institutions, agencies, and bodies in the areas of investigation and enforcement, as well as with the EU Member States, is an aspect that deserves specific consideration. This is of outmost importance since the UK is a target country for illicit tobacco—and a particularly attractive one because of the high price for licit products. Brexit will impact the current format of active participation in the work of the different agencies that operate to tackle illicit tobacco trade in the EU. Today, the UK takes part in EU police and judicial cooperation in criminal matters, including in the context of information exchange databases and systems, such as OLAF, Europol, Frontex and Eurojust. Cooperation has led to tangible results. Preserving the highest possible degree of interaction after Brexit is in the interest of both sides. The texts negotiated between the parties (the *2019 Withdrawal Agreement* and the non-binding *2019 Political Declaration*) only contain limited references to this important aspect. The institutions, agencies, and EU bodies considered, all provide for partnership mechanisms for non-EU members which, while not comparable to full membership, may offer a viable solution. Cooperation in the fight against illicit trade in tobacco products should also be fostered at the national level, with the aim of developing a comprehensive UK national anti-illicit trade strategy covering other areas than tobacco.

Finally, illicit trade in tobacco products is a global problem and addressing it requires global solutions. There is currently considerable confusion as to the post-Brexit fate of the recently introduced track and trace system mandated by TPD-2. Its efficacy is questionable, considering the current patterns of illicit trade in tobacco products, according to which most of what is consumed in the UK originates outside of the EU (where governmentally owned track and trace systems does not exist—at least for the moment). As a party in its own right to the ITP, the UK would stand to gain from the broadest possible participation in such international initiatives.

